# Comprehensive Analysis of Transcriptome-wide m^6^A Methylome Upon *Clostridium perfringens* Beta2 Toxin Exposure in Porcine Intestinal Epithelial Cells by m^6^A Sequencing

**DOI:** 10.3389/fgene.2021.689748

**Published:** 2021-10-19

**Authors:** Juanli Zhang, Qiaoli Yang, Jiaojiao Yang, Xiaoli Gao, Ruirui Luo, Xiaoyu Huang, Zunqiang Yan, Pengfei Wang, Wei Wang, Kaihui Xie, Bo Zhang, Shuangbao Gun

**Affiliations:** ^1^ College of Animal Science and Technology, Gansu Agricultural University, Lanzhou, China; ^2^ Gansu Research Center for Swine Production Engineering and Technology, Lanzhou, China

**Keywords:** m^6^A-seq, RNA-seq, beta2 toxin, M^6^A modification, porcine intestinal epithelial cells

## Abstract

Piglet diarrhea is a swine disease responsible for serious economic impacts in the pig industry. *Clostridium perfringens* beta2 toxin (CPB2), which is a major toxin of *C. perfringens* type C, may cause intestinal diseases in many domestic animals. N^6^-methyladenosine (m^6^A) RNA methylation plays critical roles in many immune and inflammatory diseases in livestock and other animals. However, the role of m^6^A methylation in porcine intestinal epithelial (IPEC-J2) cells exposed to CPB2 has not been studied. To address this issue, we treated IPEC-J2 cells with CPB2 toxin and then quantified methylation-related enzyme expression by RT-qPCR and assessed the m^6^A methylation status of the samples by colorimetric N^6^-methyladenosine quantification. The results showed that the methylation enzymes changed to varying degrees while the m^6^A methylation level increased (*p* < 0.01). On this basis, we performed N^6^-methyladenosine sequencing (m^6^A-seq) and RNA sequencing (RNA-seq) to examine the detailed m^6^A modifications and gene expression of the IPEC-J2 cells following CPB2 toxin exposure. Our results indicated that 1,448 m^6^A modification sites, including 437 up-regulated and 1,011 down-regulated, differed significantly between CPB2 toxin exposed cells and non-exposed cells (*p* < 0.05). KEGG pathway analysis results showed that m^6^A peaks up-regulated genes (*n* = 394) were mainly enriched in cancer, Cushing syndrome and Wnt signaling pathways, while m^6^A peaks down-regulated genes (*n* = 920) were mainly associated with apoptosis, small cell lung cancer, and the herpes simplex virus 1 infection signaling pathway. Furthermore, gene expression (RNA-seq data) analysis identified 1,636 differentially expressed genes (DEGs), of which 1,094 were up-regulated and 542 were down-regulated in the toxin exposed group compared with the control group. In addition, the down-regulated genes were involved in the Hippo and Wnt signaling pathways. Interestingly, the combined results of m^6^A-seq and RNA-seq identified genes with up-regulated m^6^A peaks but with down-regulated expression, here referred to as “hyper-down” genes (*n* = 18), which were mainly enriched in the Wnt signaling pathway. Therefore, we speculate that the genes in the Wnt signaling pathway may be modified by m^6^A methylation in CPB2-induced IPEC-J2 cells. These findings provide new insights enabling further exploration of the mechanisms underlying piglet diarrhea caused by CPB2 toxin.

## Introduction

*Clostridium perfringens*, which is one of the most important zoonotic pathogens (Immerseel et al., 2005), can cause severe bleeding ulcers and mucosal necrosis of the small intestine in humans and animals. It can produce four main toxins (α, β, ε, ι), according to which it is divided into five types: A, B, C, D, and E (Garcia et al., 2012). The *C. perfringens* beta2 (CPB2) toxin was first discovered in the supernatant of a culture *of C. perfringens* type C by Gibert et al. (1997). Its protein molecular weight is 28 kDa, with an isoelectric point of 5.4*–*5.5, and it is associated with gastrointestinal diseases in livestock caused by *C. perfringens*. The effect of CPB2 toxin on intestinal membrane epithelial cells indicates that it is strongly cytotoxic. When a low concentration of the toxin is introduced into these cells, the cells become rounded and damaged (Gibert et al., 1997). However, the mechanism by which CPB2 toxin induces diarrhea in animals (especially pigs) is still unclear and should be further studied.

N^6^-methyladenosine (m^6^A) modification is pervasive within mRNA and plays important regulatory roles in various biological processes (Meyer et al., 2012). In mammalian species, about one-third of mRNAs are modified by m^6^A (average of three to five m^6^A modifications in each mRNA), and many m^6^A sites are evolutionarily conserved between humans and mice (Huang et al., 2020). Dominissini et al. (2012) used m^6^A-specific antibodies to enrich fragments containing m^6^A and to identify the expression of m^6^A in whole transcripts of humans and mice by a high-throughput sequencing method (MeRIP-seq). m^6^A is mainly located near the termination codons and 3′UTRs of mRNA, and can influence pre-mRNA splicing (Zhao et al., 2014; Xiao et al., 2016), RNA structure (Liu et al., 2015), nuclear mRNA (Fustin et al., 2013), miRNA maturation (Alarcón et al., 2015), mRNA stability (Huang et al., 2018), chromosome inactivation (Patil et al., 2016), mRNA translation (Shi et al., 2017), and even RNA degradation (Zhu et al., 2014).

m^6^A modification plays very important roles in various immune and inflammatory responses to bacterial infection, such as the inflammatory response of human dental pulp cells (HDPCs) induced by lipopolysaccharide (LPS) (Feng et al., 2018) and the response of porcine small intestinal epithelial (IPEC-J2) cells to *Escherichia coli* K88 infection (Zong et al., 2018). In addition, Wu et al. (2020) found that deletion of YTH domain family 2 (YTHDF2) in the m^6^A reader promotes the demethylation of histone H3 lysine-27 trimethylation (H3K27me3) modifications, which in turn increases proinflammatory cytokine levels. However, the role of m^6^A modification in IPEC-J2 cells exposed to CPB2 remains unclear. Therefore, to better understand m^6^A modification in response to CPB2 toxin exposure, we performed N^6^-methyladenosine sequencing (m^6^A-seq) and RNA sequencing (RNA-seq) to investigate differentially methylated genes (DMGs) and differentially expressed genes (DEGs) in IPEC-J2 cells that were exposed to CPB2. Our results provide a theoretical basis for further research into the molecular mechanisms of m^6^A modification in IPEC-J2 cells exposed to CPB2.

## Materials and Methods

### CPB2 Toxin Extraction and Purification

The extraction and purification of CPB2 toxin were performed according to the method described by Luo et al. (2020). Briefly, a recombinant plasmid containing the CPB2 gene was successfully constructed and transformed into *E. coli* BL21 competent cells. Then, a single colony was selected, placed in medium containing kanamycin and incubated for 2.5 h at 37°C and 220 rpm. When the optical density (OD) reached 0.5–0.8, 1 mM isopropyl β-d-1-thiogalactopyranoside (IPTG, Solarbio, Beijing, China) was added to induce the bacteria at 16°C for 12 h. The precipitates were collected and suspended in 20 mM Tris-HCl buffer (pH 8.0), sonicated on ice, and purified by using High-Affinity Ni-Charged Resin FF (GenScript, Nanjing, China). The resin was washed with 10 mM imidazole lysis buffer and then eluted with 250 and 500 mM imidazole. Recombinant protein expression was detected by 12% alkyl sulfate-polyacrylamide gel electrophoresis (SDS-PAGE). Next, 10 mL of protein sample was loaded into a membrane and purified by dialysis against 1 × PBS (pH 7.6) for 24 h, then concentrated with PEG6000 for 40 min. Finally, Endotoxin Erasol (Genscript, Nanjing, China) was used to remove endotoxins.

### Cell Culture

IPEC-J2 cells were provided by the Beijing Beina Chuanglian Institute of Biotechnology (Beijing, China) and cultured in 90% Dulbecco’s modified Eagle’s medium (DMEM, HyClone, Logan, United States) with 10% fetal bovine serum (FBS, HyClone), penicillin (100 U/mL), and streptomycin (100 μg/mL) at 37°C in a 5% CO_2_ atmosphere. The medium was changed every 2 days, and the cells were passaged by trypsinization at 80–90% confluency.

### CPB2 Toxin Treatment of Cells and Total m^6^A Measurement

IPEC-J2 cells were seeded in 6-well plates at a density of 1×10^5^ cells/mL and cultured overnight (24 h). Then, cells in three wells were each treated with 20 μg/mL CPB2 (treatment group) while cells in the other three wells were not exposed to toxin (control group) (Gao et al., 2020; Luo et al., 2020). Total RNA was extracted by using TRIzol reagent according to the manufacturer’s instructions (Invitrogen, Carlsbad, United States), and the m^6^A content was detected with an m^6^A RNA methylation quantification kit (Epigentek, Farmingdale, United States). Specifically, RNA purity was confirmed according to the following ratios: A260/A280 > 1.9 and A260/A230 > 1.7. In each reaction, the RNA (200 ng) samples were treated with an m^6^A antibody, and detection was performed based on the manufacturer’s instructions. Finally, the absorbance values were measured at 450 nm.

### Real-Time Quantitative PCR (RT-qPCR)

RNA (800 ng) from each sample was employed to synthesize cDNA by using a reverse transcription kit (Accurate Biotechnology, Changsha, China). Thereafter, a SYBR^®^ Green Premix Pro Taq HS qPCR Kit (Accurate Biotechnology) and a LightCycler 480II apparatus (Roche, Basel, Switzerland) were used to perform RT-qPCR. The sequences of the primers used in this study are presented in Table 1; *GAPDH* served as an internal control. The relative levels of DEGs were determined by the 2^−ΔΔCt^ method (Livak and Schmittgen, 2001).

**TABLE 1:** Primer sequences for RT-qPCR and MeRIP-qPCR.

Gene	Primer sequence (5′-3′)	Product length (bp)	Accession no
*METTL3*	F: CCA​CTT​CTG​GTG​GCC​CTA​AG	104	XM_003128580.5
	R: CGC​CAG​ATC​AGA​AAG​GTG​GT		
*METTL14*	F: GAG​ATT​GCA​GCT​CCT​CGA​TCA	89	XM_003129231.6
	R: CCC​CAC​TTG​CGT​AAA​CAC​AC		
*WTAP*	F: GCG​GGA​ATA​AGG​CCT​CCA​AC	136	XM_005659114.3
	R: TGT​GAG​TGG​CGT​GTG​AGA​GA		
*FTO*	F: GCA​TGG​CTG​CTT​ATT​TCG​GG	154	NM_001112692.1
	R: TGC​ATC​AGA​GCC​CTT​CAC​TG		
*ALKBH5*	F: CCA​GTT​CAA​GCC​TAT​CCG​GG	80	XM_021067995.1
	R: ATC​CAC​TGA​GCA​CAG​TCA​CG		
*YTHDF1*	F: ATC​GCC​TCC​TAC​AAG​CAC​TC	111	XM_021078236.1
	R: CTG​TTT​GCT​CCG​ATT​CTG​CC		
*YTHDF2*	F: GGA​CAA​CTG​AGC​AAC​GGA​GA	131	XM_005665152.3
	R: GCT​GAG​AAG​TCA​ATC​CCG​CT		
*YTHDF3*	F: CAA​CCA​ATA​GTG​TGC​CCC​CA	214	XM_021089309.1
	R: TGG​GTT​GGT​GGA​GCC​TTT​AC		
*YTHDC1*	F: TGA​GAA​TGT​GTC​CCT​TGC​CA	230	XM_021101402.1
	R: CAC​CTC​CCA​GCA​TCT​TAG​CAT		
*YTHDC2*	F: GAA​GTG​ATG​GAT​GGG​AGC​GA	142	XM_021084629.1
	R: ATA​TCA​CCA​CCA​CCT​CGT​GC		
*EGR1*	F: GGA​CAT​GGC​GAC​AAC​CTT​TT	139	XM_003123974.6
	R: TCC​CAC​CTA​AGA​GGA​ACC​CT		
*MYC*	F: TCA​GAA​AAA​GAC​GTG​CTG​CG	102	NM_001005154.1
	R: AGT​TCC​TCC​CTC​CAA​TAG​GTC​A		
*FZD7*	F: CAA​GTT​CGG​CTT​CCA​ATG​GC	148	XM_013984388.2
	R: CAT​GTA​GGG​CGC​TGT​AGG​AT		
*WNT9A*	F: GGA​GAA​GAA​CTG​CGA​GAG​CAT	141	XM_003123611.5
	R: GTA​TAG​ACC​TCC​TCA​CGC​TGG		
*FOSL1*	F: CCC​CAG​TGG​AAG​TGG​TTC​AG	137	XM_003122519.3
	R: GAA​GTC​TCG​GAA​CAT​GCC​CT		
*ITGA9*	F: AAG​ACA​GTT​GGG​ACT​GGG​TC	141	XM_021071659.1
	R: AGT​GGG​GCC​TCC​GAA​AAA​TC		
*IL2RA*	F: GCA​ACC​ATG​CAG​CCA​ATC​AT	161	XM_021064060.1
	R: GGC​TTC​TTA​CTG​CCC​TTG​GT		
*TLR2*	F:GGAGCCTTAGAAGTAGAGTTTG	102	NM_213761.1
	R: TGT​CTC​CAC​ATT​ACC​GAG​GG		
*FZD5*	F: TCG​TGA​GGC​CAT​TAC​TGG​GA	94	XM_0056722133.3
	R: TTT​CGG​CTT​CTC​AAA​TGC​GG		
*WNT11*	F: CAT​GCG​CTT​CGC​TTC​CAC​TT	87	XM_013979149.2
	R: TCG​GCT​TCC​TTT​GAT​GTC​CTG		
*GAPDH*	F: AGT​ATG​ATT​CCA​CCC​ACG​GC	139	XM_021091114.1
	R: TAC​GTA​GCA​CCA​GCA​TCA​CC		

### m^6^A-Seq and RNA-Seq Library Construction

First, RNA was extracted, and the RNA purity and integrity were analyzed. Total RNA was extracted from IPEC-J2 cells (see *CPB2 toxin treatment of cells and total m6A measurement*) by using TRIzol reagent (Invitrogen, Carlsbad, United States) and then quantified with a NanoDrop ND-1000 instrument (NanoDrop, Wilmington, United States). A Bioanalyzer 2100 system (Agilent, Santa Clara, United States) was used to evaluate the integrity of RNA according to the criterion of an RNA integrity number (RIN) > 7.0. RNA integrity was further confirmed by denaturing agarose gel electrophoresis. Second, the input (RNA-seq) library and the immunoprecipitation (IP) (m^6^A-seq) library were constructed. Briefly, an Epicenter Ribo-Zero Gold Kit (Illumina, San Diego, United States) was applied to digest and remove ribosomal RNA (rRNA) from the total RNA. A Magnesium RNA Fragmentation Module (NEB, Ipswich, United States) was used to fragment the RNA into 150-nucleotide-long fragments at 86°C for 7 min. The obtained RNA fragments were then divided into two portions. One portion was used to directly construct a conventional transcriptome sequencing library, which was retained as the input RNA, while the other portion was enriched by an m^6^A-specific antibody (Synaptic Systems, Gottingen, Germany) and was retained as IP RNA. Thereafter, the IP RNA and input RNA were reverse-transcribed to cDNA by SuperScript™ II Reverse transcriptase (Invitrogen, Carlsbad, United States). The second strand of DNA was then synthesized, and adapters were added to the blunt ends of each strand (each adapter contained a T-base overhang for ligating the adapter to the A-tailed fragmented DNA) by using *E. coli* DNA polymerase I (NEB, Ipswich, United States), RNase H (NEB, Ipswich, United States) and a dUTP Solution (Thermo Fisher, San Jose, United States). Then, the two strands were digested with the enzyme UDG (NEB), after which PCR was conducted with the following program: predenaturation at 95°C for 3 min; eight cycles of 15 s at 98°C, 15 s at 60°C (annealing), and 30 s at 72°C (extension); and a final extension at 72°C for 5 min. The average insert size for the final cDNA library was 300 ± 50 bp. Finally, the Illumina NovaSeq™ 6000 platform (Illumina, San Diego, United States) was used to carry out double-ended sequencing according to its standard operating procedures in PE150 sequencing mode by LC Bio Technology Co., Ltd., Hangzhou, China.

### Bioinformatic Analysis of m^6^A-Seq and RNA-Seq

Data quality control. We handled the raw data reads of the IP and input samples by using Fastp software (https://github.com/OpenGene/fastp). In this process, the reads showing adaptor contamination, presenting an N ratio >5%, and containing low-quality sequences were removed. Finally, clean reads were obtained (Chen et al., 2018; Wang et al., 2021; Zhao et al., 2021).

Peak identification and differential peak analysis. HISAT2 software (Kim et al., 2015) (http://daehwankimlab.github.io/hisat2) was used to map reads to the *Sus scrofa* 11.1 reference genome (ftp://ftp.ensembl.org/pub/release-96/fasta/sus_scrofa/dna/). The mapped reads of the IP and input samples were employed for differential peak analysis by using the R package exomePeak (https://bioconductor.org/packages/exomePeak) (Meng et al., 2014; Wang et al., 2021). The peaks were visualized with IGV software (http://www.igv.org) (Robinson et al., 2017; Yang et al., 2020). We annotated the peaks by using ChIPseeker (https://bioconductor.org/packages/ChIPseeker) (Yu et al., 2015). Finally, a motif analysis was performed with the MEME2 (http://meme-suite.org) (Bailey et al., 2009) and HOMER (http://homer.ucsd.edu/homer/motif) (Heinz et al., 2010) softwares.

DMGs and DEGs analysis. StringTie (https://ccb.jhu.edu/software/stringtie) was used to determine the expression levels of all the mRNA transcripts from the input libraries by calculating their fragments per kilobase of transcript per million mapped reads (FPKM) values (total exon fragments/mapped reads [millions] × exon length [kb]) (Pertea et al., 2015), and the R package edgeR (https://bioconductor.org/packages/edgeR) was used to analyze the DEGs (Robinson et al., 2010). Gene Ontology (GO) and Kyoto Encyclopedia of Genes and Genomes (KEGG) analyses were performed with Database for Annotation, Visualization and Integrated Discovery by using OmicStudio tools (https://www.omicstudio.cn/tool) (Chen et al., 2020; Zhou et al., 2020).

### M^6^A IP (MeRIP) Followed RT-qPCR (MeRIP-qPCR)

RNA (1,000 ng/μL) samples from the control and CPB2 groups (300 μL each sample) were subjected to m^6^A IP using the GenSeq^®^ m^6^A MeRIP Kit (GenSeq Inc., Shanghai, China) according to the manufacturer’s protocol (Wang et al., 2020a; Hou et al., 2021). Briefly, the RNA was fragmented into smaller pieces of 200 nt by using an RNA fragmentation reagent and then divided into 3 and 297 μL. 3 μL was directly reverse-transcribed into cDNA (input), while the 297 μL was enriched with an m^6^A antibody and then reverse-transcribed into cDNA (IP) after purification. Finally, RT-qPCR was performed for detection.

### Statistical Analyses

All experimental procedures were performed at least three times. The m^6^A peaks were filtered according to two criteria: enrichment ≥2 and a *p*-value < 0.05. The differential peaks and DEGs were screened in the CPB2 group vs the control group according to a |log2 (fold change)| >0.585 and a *p*-value < 0.05. The RT-qPCR and MeRIP-qPCR data were analyzed by using the SPSS v.21 and GraphPad Prism v.8.0 software programs. The mean ± standard deviation (SD) was used to describe the data, and statistically significant differences are denoted with one asterisk (*) for *p* < 0.05 or two asterisks (**) for *p* < 0.01.

## Results

### Detection of Methylation-Related Enzyme Expression and m^6^A Levels

**FIGURE 1:**
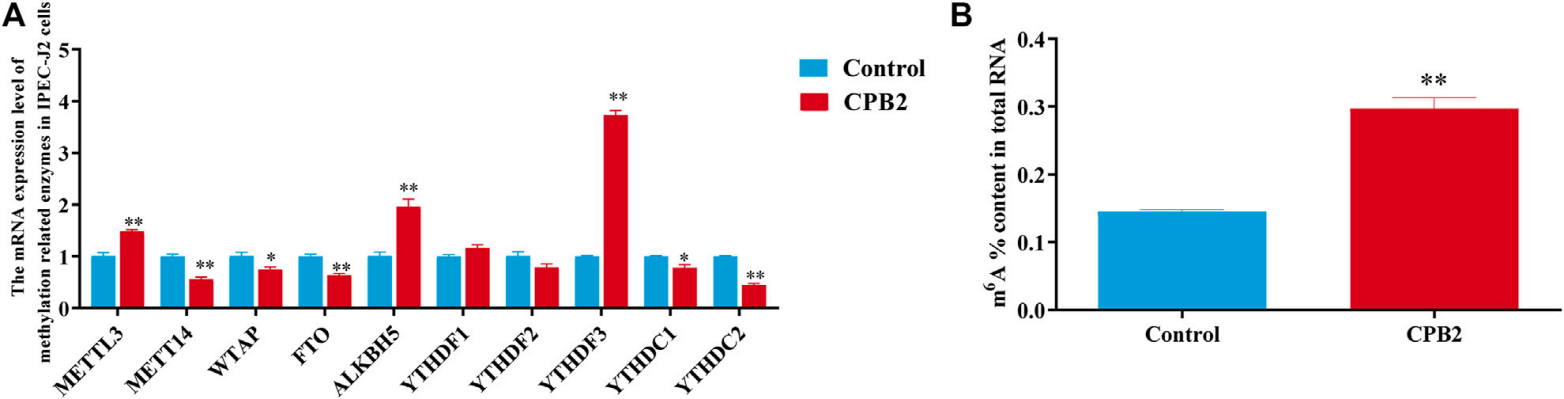
Evaluation of the expression levels of methylase-related enzymes and m^6^A levels under 20 μg/mL CPB2 treatment. **(A)** qPCR analysis of the expression of methylation-related enzymes after 24 h of cells exposure to 20 μg/ml CPB2 toxin. **(B)** Determination of m^6^A levels in IPEC-J2 cells after treating with 20 μg/mL CPB2 for 24 h.

To investigate the effect of m^6^A modification in CPB2-induced IPEC-J2 cells, the mRNA expression levels of methylation-related enzymes and the total m^6^A content of the control and CPB2 groups were detected by RT-qPCR and with an m^6^A RNA methylation quantification kit, respectively. As shown in Figures 1A,B, the expression levels of *METTL3*, *ALKBH5*, and *YTHDF3* were increased dramatically (*p* < 0.01), while those of *METTL14*, *FTO*, and *YTHDC2* were decreased significantly in the CPB2 toxin group relative to the control group (*p* < 0.01); *WTAP* and *YTHDC1* levels were also decreased (*p* < 0.05), but *YTHDF1* and *YTHDF2* levels were not changed (*p* > 0.05) (Figure 1A)*.* In addition, the total m^6^A level was obviously increased in the CPB2 group (*p* < 0.01, Figure 1B). These results suggested that differences in m^6^A methylation modifications may exist between the control group and the CPB2 group.

### Sequence Statistics and Quality Control

To further explore the biological process of m^6^A modification in IPEC-J2 cells exposed to CPB2 toxin, three samples each from the control and CPB2 groups were collected for m^6^A sequencing. Two types of libraries corresponding to the control group and CPB2 group were further constructed and designated the IP libraries (m^6^A-seq) and the input libraries (RNA-seq), respectively. For the IP libraries, a total of 89,642,446–90,242,216 raw reads were obtained for the CPB2 group, while 85,492,316–87,774,992 raw reads were obtained for the control group (Table 2). For the input libraries, there were 92,752,804–97,478,686 raw reads for the CPB2 group and 93,495,320–97,586,308 raw reads for the control group (Table 2). The percentages of clean (valid) reads for the control group and the CPB2 group were greater than 86.79% in the IP libraries and 83.24% in the input libraries. For all libraries, the Q20 and Q30 values were at least 90% (Table 2 and Supplementary Table S1). In addition, clean reads whose ribosome sequences were removed were compared with the porcine reference genome. The results showed that the clean reads were mainly aligned to exon regions, introns, and intergenic regions, and the proportions of exons exceeded 60% in the control and CPB2 groups (Supplementary Figure S1; Table S2).

**TABLE 2:** Sequence statistics and quality control.

Sample ID	Raw reads	Valid reads	Valid%	Q20%	Q30%	GC%
Control1_IP	85,492,316	82,300,032	87.19	96.52	90.94	52.87
Control2_IP	87,774,992	84,673,108	88.09	96.52	90.97	53.28
Control3_IP	86,612,186	83,480,924	87.76	96.33	90.59	53.39
CPB2_1_IP	90,242,216	86,944,982	87.8	96.56	91.01	52.96
CPB2_2_IP	86,934,932	83,429,868	86.82	96.39	90.78	53.41
CPB2_3_IP	89,642,446	86,070,422	86.79	96.41	90.77	53.99
Control1_input	97,586,308	93,048,292	84.9	96.54	91.09	55.12
Control2_input	96,994,146	92,000,280	84.62	96.53	91.09	55.3
Control3_input	93,495,320	88,512,112	84.2	96.3	90.67	55.92
CPB2_1_input	95,043,768	90,159,134	84.15	96.58	91.17	55.18
CPB2_2_input	97,478,686	91,769,152	83.24	96.37	90.85	55.73
CPB2_3_input	92,752,804	88,474,096	84.76	96.46	90.95	55.71

### Peak Distributions of m^6^A Modifications in the Whole Genome

**FIGURE 2:**
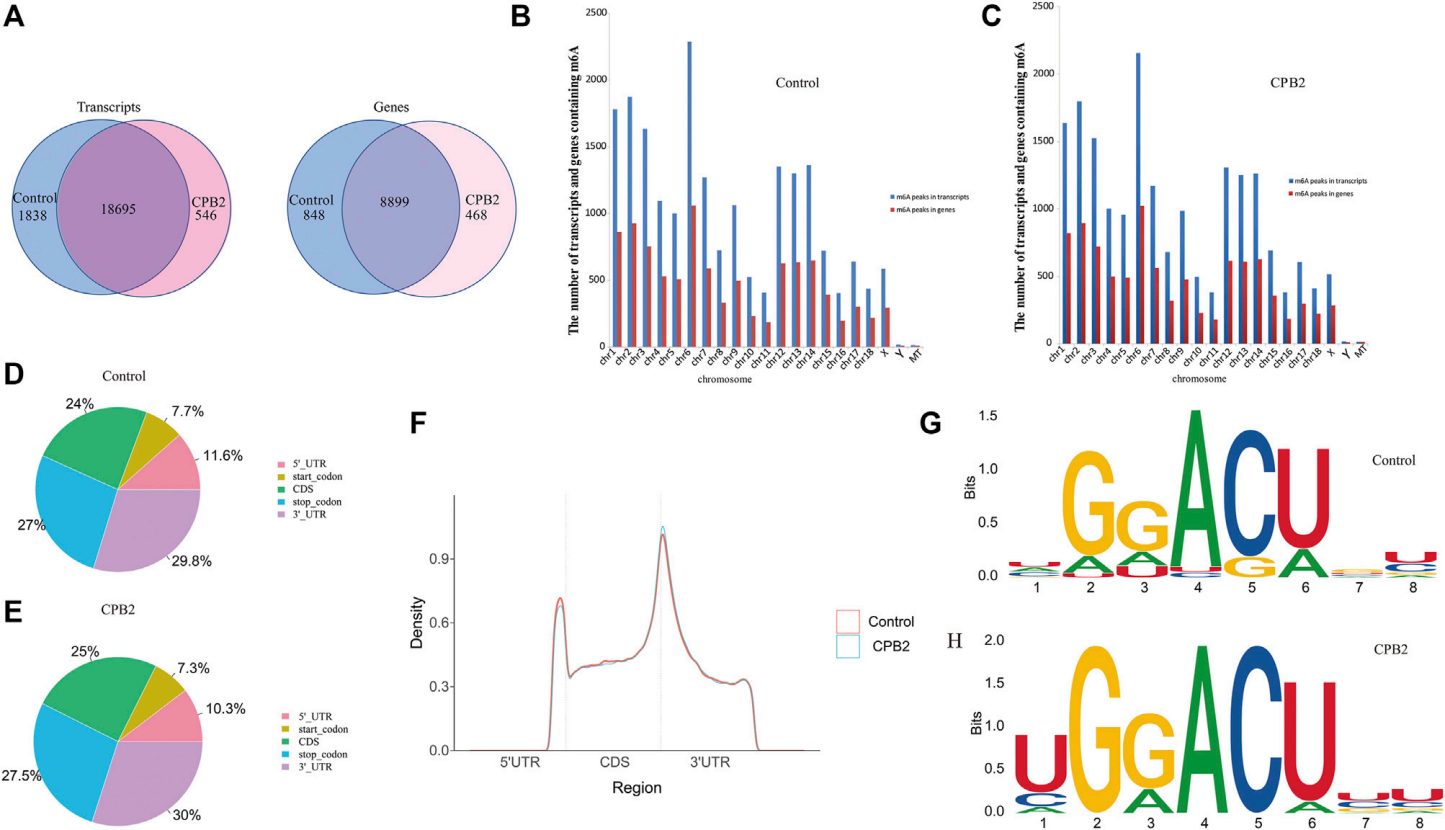
Peak Distribution. **(A)** m^6^A peaks distributions in the control group and CPB2 group among mRNA transcripts (left) and genes (right). **(B)** Numbers of m^6^A peaks in the mRNA transcripts and genes of chromosomes in the control group. **(C)** Numbers of m^6^A peaks in the mRNA transcripts and genes of chromosomes in the CPB2 group. **(D)** Distributions of m^6^A peaks in different gene functional elements (5′UTR, 3′UTR, 1st exon, and other exons) in the control group. **(E)** Distributions of m^6^A peaks in different gene functional elements (5′UTR, 3′UTR, 1st exon, and other exons) in the CPB2 group. **(F)** Density distributions of m^6^A peaks in different gene functional elements (5′UTR, CDS, and 3′UTR) in the control group and CPB2 group. **(G, H)** Motif sites in the control (top) and CPB2 groups (bottom).

After the full-range peak calling results of the porcine reference genome were scanned, a Poisson distribution model was used to check the reads of the candidate peak regions and to calculate the *p*-values of given peak regions. In this experiment, we were able to distinguish the differences in these peaks (i.e., enrichment ≥2, *p* < 0.05) and identify the peaks in the IP libraries, whose results were compared with those of the input libraries. We found that the numbers of peaks in the control group vs the CPB2 group were 20,533 and 19,241, respectively (Supplementary Table S3). Additionally, 18,695 peaks were the same between the control group and the CPB2 group, accounting for 90% of all detected peaks (Figure 2A). Furthermore, we analyzed the distributions of peaks on the pig chromosomes in the CPB2 group and control group. Interestingly, there were more peaks on chromosomes 1, 2, 3, 6, 12, 13, and 14 than on other chromosomes, and the most peaks were distributed on chromosome 6 (Figures 2B,C, Supplementary Table S4). Then, we analyzed the distributions of peaks in gene functional elements in both groups. The results showed that the m^6^A peaks in the control group and CPB2 group were mainly enriched in the 3′UTRs and stop codon regions (Figures 2D,E, Figure 2F). Next, HOMER and MEME2 softwares were employed to identify the most reliable motifs in the peak regions, which identified the GGACU motif in the control group and the UGGACU motif in the CPB2 group (Figures 2G,H).

### Differential m^6^A Peaks Between the CPB2 and Control Groups

**FIGURE 3:**
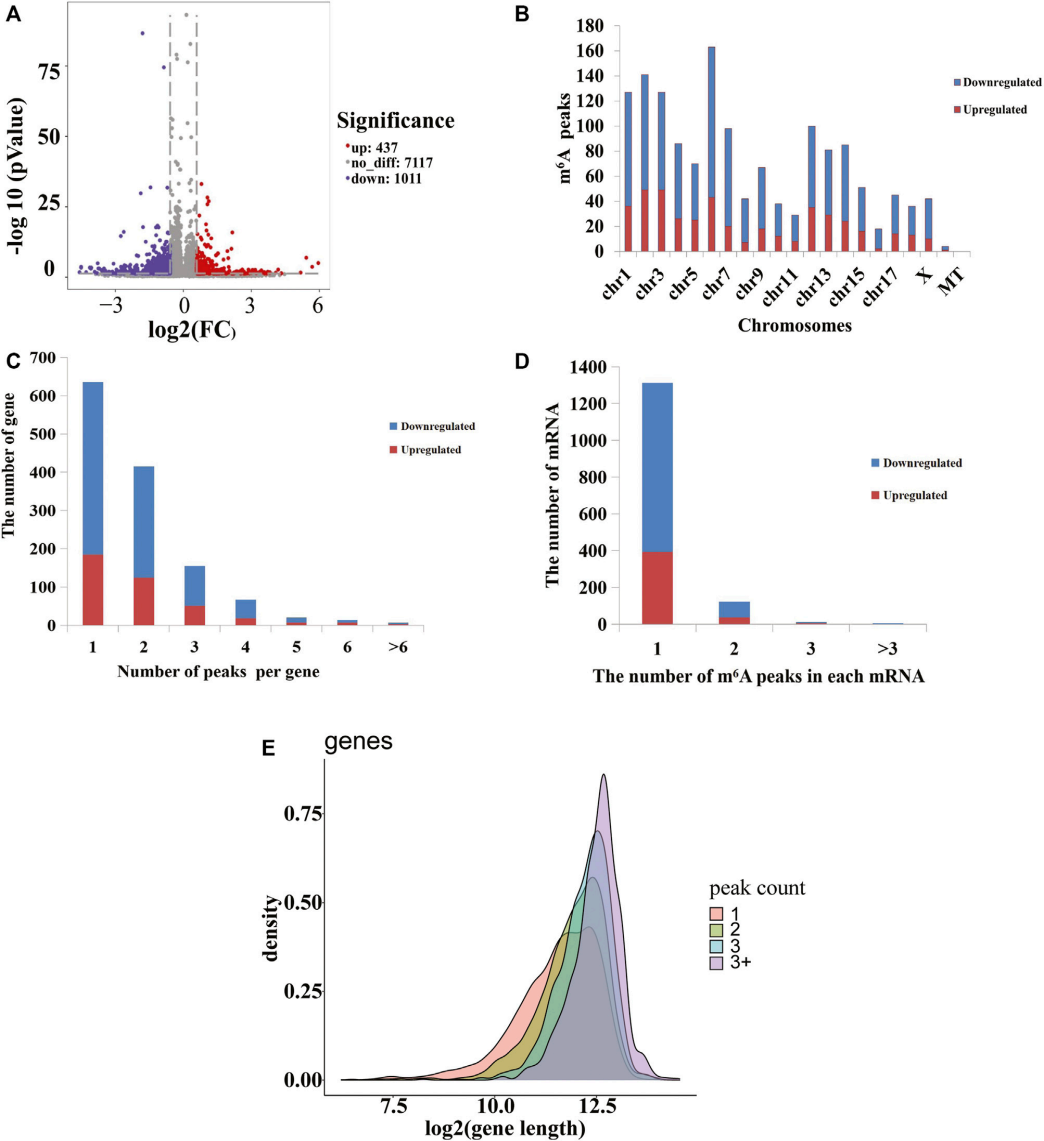
m6A peaks in the control and CPB2 groups. **(A)** Significantly different m^6^A peaks in the CPB2 group vs the control group. **(B)** Significant differences in the distributions of m^6^A peaks on pig chromosomes. **(C)** Distributions of differential m^6^A peaks in per gene. **(D)** Distributions of differential m^6^A peaks in per mRNA. **(E)** Distributions of peaks with different sizes.

To analyze the distributions of m^6^A peaks among the different chromosomes, genes, and mRNA transcripts in the CPB2 and control groups, we scanned the differential peaks and found 1,448 differential m^6^A peaks. Among these peaks, we found 437 significantly upregulated peaks in 394 genes (here referred to as differentially methylated genes, DMGs) and 1,011 significantly downregulated peaks in 920 genes (Figure 3A and Supplementary Table S5). The top 20 altered m^6^A peaks represented the 10 most upregulated genes and the 10 most downregulated genes (Table 3 and Supplementary Table S5). Furthermore, relative to the control group (excluding the peaks showing no significant difference on the mitochondrial chromosome), the CPB2 group exhibited differential peaks distributed on other chromosomes; there were more downregulated peaks than upregulated peaks, and the peaks were most abundant on chromosome 6 (Figure 3B and Supplementary Table S6). We also counted the peaks distributed in genes and found that many genes contained one peak, which accounted for 46.7% (184/394) of the DMGs with upregulated peaks and 48.9% (452/920) DMGs with downregulated (Figure 3C and Supplementary Table S7). In analyzing the number of differential peaks among all mRNA transcripts, we found that the majority of mRNA transcripts in the whole genome showed one peak, while a few mRNA transcripts showed two or more differential peaks (Figure 3D and Supplementary Table S7). In addition, we calculated the densities of m^6^A modifications among mRNA molecules and found that the gene fragments containing more m^6^A peaks were longer (Figure 3E).

**TABLE 3:** Top 10 significantly upregulated and top 10 significantly downregulated m^6^A peaks (CPB2 vs control).

Chromosome	Peak start	Peak end	Gene ID	Gene name	Regulation	*p*-value	Log2 (fold change)	Peak region
chr12	41,074,651	41,075,895	ENSSSCG00000031620	ENSSSCG00000031620	up	8.91E-06	5.95	3′UTR
chr3	10,755,262	10,755,381	ENSSSCG00000023004	FZD9	up	0.00020417	5.68	CDS
chr18	6,382,439	6,383,066	ENSSSCG00000033909	ENSSSCG00000033909	up	1.20E-07	5.42	CDS
chr9	121,457,341	121,457,431	ENSSSCG00000033352	FAM163A	up	0.02238721	5.17	3′UTR
chr10	28,509,561	28,509,920	ENSSSCG00000033321	GAS1	up	0.03890451	4.32	CDS
chr15	140,319,882	140,320,272	ENSSSCG00000034101	D2HGDH	up	0.00194984	4.18	CDS
chr2	70,622,697	70,622,965	ENSSSCG00000013607	ENSSSCG00000013607	up	0.02570396	4.07	5′UTR
chr3	113,558,282	113,558,342	ENSSSCG00000029413	DNMT3A	up	0.01659587	3.81	CDS
chr4	98,093,837	98,093,896	ENSSSCG00000006634	TNFAIP8L2	up	0.03311311	3.81	3′UTR
chr8	12,807,001	12,807,091	ENSSSCG00000008747	NCAPG	up	0.01202264	3.72	3′UTR
chrY	25,251,102	25,252,331	ENSSSCG00000034853	ENSSSCG00000034853	down	0	-10.1	3′UTR
chr6	54,469,817	54,469,877	ENSSSCG00000038579	DKKL1	down	0.01737801	-4.58	3′UTR
chr4	97,240,279	97,240,459	ENSSSCG00000006610	S100A11	down	0.00019953	-4.51	3′UTR
chr7	52,922,051	52,922,196	ENSSSCG00000035897	ENSSSCG00000035897	down	0.00041687	-4.51	CDS
chr7	23,655,139	23,655,378	ENSSSCG00000001400	DDX39B	down	0.01659587	-4.29	3′UTR
chr7	36,352,159	36,352,488	ENSSSCG00000038553	TSPO2	down	0.00038905	-4.13	3′UTR
chr4	99,142,708	99,142,917	ENSSSCG00000006666	SV2A	down	0.00091201	-4.08	5′UTR
chrX	58,487,996	58,488,554	ENSSSCG00000011830	ENSSSCG00000011830	down	0.00039811	-4.06	CDS
chrY	25,253,141	25,253,380	ENSSSCG00000034853	ENSSSCG00000034853	down	0.01230269	-3.91	3′UTR
chr6	159,511,892	159,511,981	ENSSSCG00000027119	SELRC1	down	0.03311311	-3.81	5′UTR

### GO and KEGG Analysis of Genes Presenting Differential m^6^A Peaks (DMGs)

**FIGURE 4:**
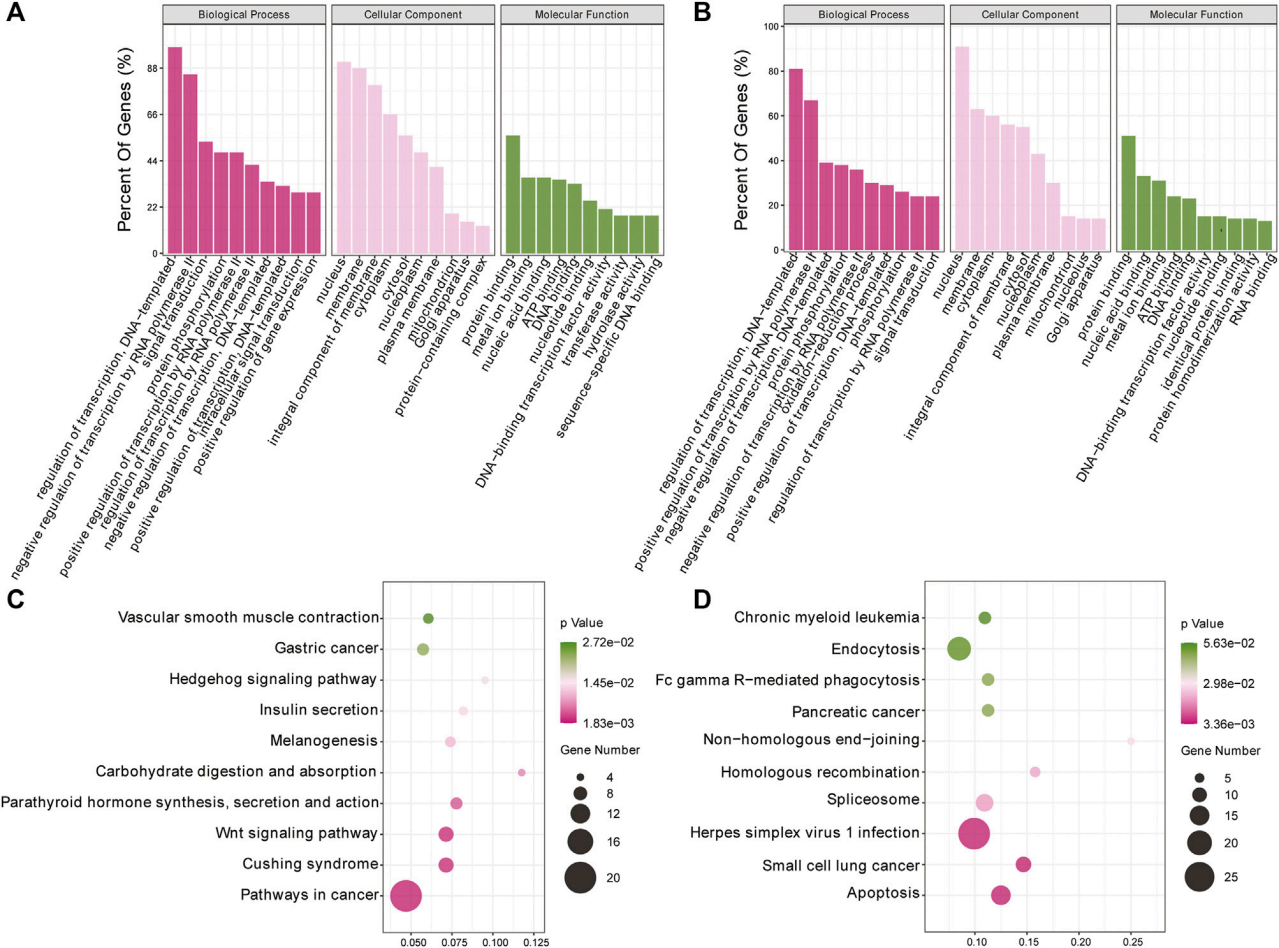
GO and KEGG analyses of DMGs. **(A, B)** Top 10 GO terms of the differentially methylated **(A)** upregulated genes and **(B)** downregulated genes. **(C, D)** Top 10 enriched KEGG pathways of the differentially methylated **(C)** upregulated genes and **(D)** downregulated genes.

To investigate the biological processes associated with m^6^A modification in IPEC-J2 cells in response to CPB2 infection, we analyzed the functions of DMGs presenting upregulated m6A peaks (n = 437) or downregulated peaks (n = 1,011) according to GO terms and KEGG signaling pathways. The GO results were divided into three categories: the biological process (BP), cellular component (CC), and molecular function (MF) categories. The top 10 BP, CC, and MF terms that were enriched for genes with upregulated and downregulated m^6^A peaks are shown in Figures 4A,B. For the KEGG pathway analysis, we display the top 10 enriched KEGG pathways based on their *p*-values. The results showed that genes with upregulated m^6^A peaks were mainly enriched in cancer signaling pathways (20 genes), the Cushing syndrome signaling pathway (9 genes), and the Wnt signaling pathway (9 genes) (Figure 4C and Supplementary Table S5), while genes with downregulated m^6^A peaks were mainly enriched in the apoptosis pathway (15 genes), the small cell lung cancer signaling pathway (11 genes) and the herpes simplex virus 1 infection signaling pathway (27 genes) (Figure 4D and Supplementary Table S5).

### DEGs (RNA-Seq Data) Following CPB2 Treatment of IPEC-J2 Cells

**FIGURE 5:**
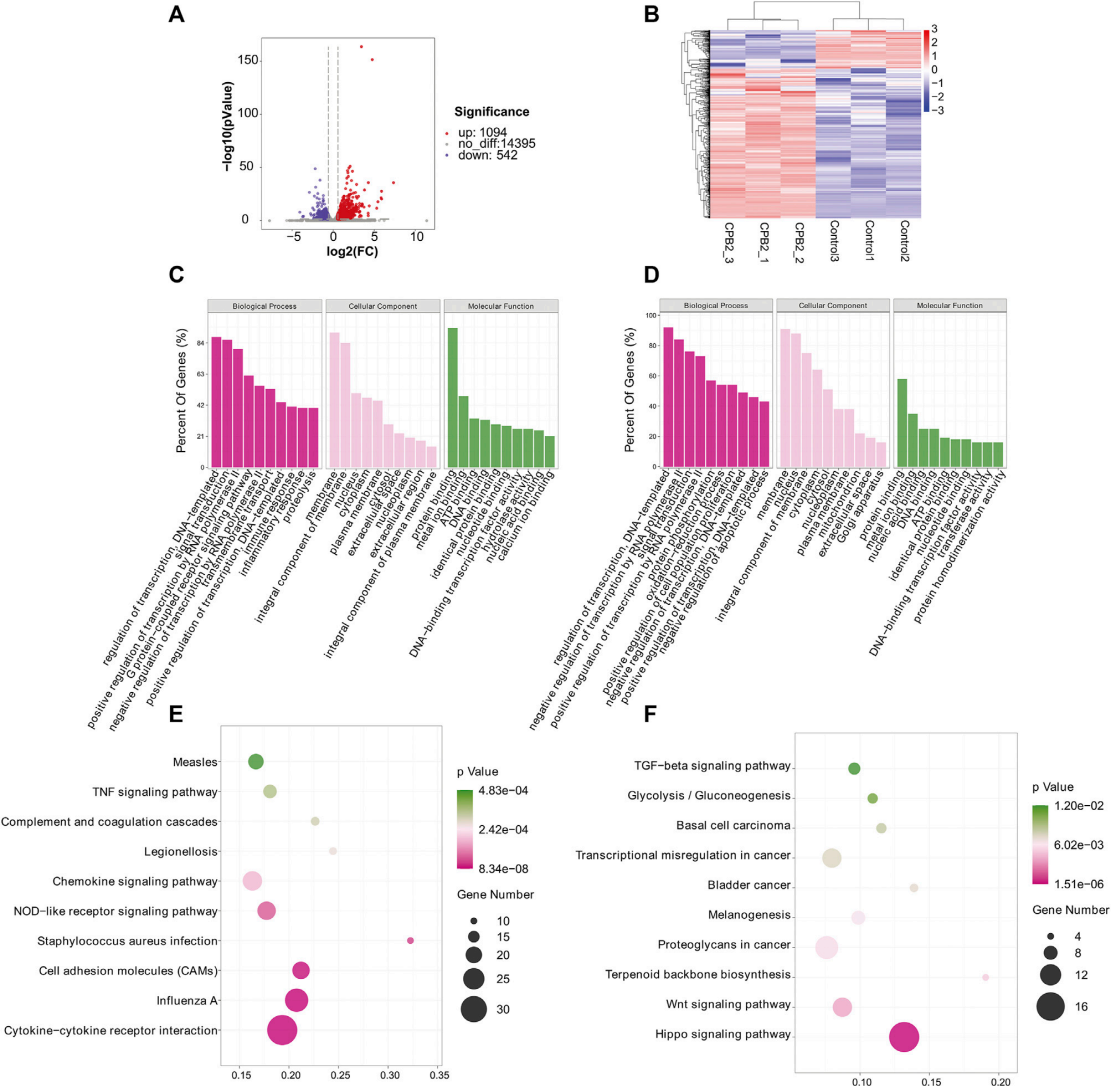
GO and KEGG analyses of DEGs. **(A)** Volcano plot showing the differential gene expression in the CPB2 and control groups. **(B)** Heatmap showing the overall expression patterns of DEGs in three CPB2 individuals and three control individuals. Red indicates upregulated DEGs, and blue indicates downregulated DEGs. **(C, D)** Top 10 GO terms of the (C) upregulated and (D) downregulated DEGs. **(E, F)** Top 10 KEGG pathways enriched for the (E) upregulated and (F) downregulated DEGs.

We used RNA-seq data (the m^6^A-seq input library) to analyze DEGs after treating IPEC-J2 cells with CPB2 (Figures 5A,B, Supplementary Table S8). In total, 1,636 DEGs were detected in the CPB2 group relative to the control group, among which 1,094 DEGs were upregulated and 542 DEGs were downregulated. We list the top 20 most significantly upregulated genes and downregulated genes in tables (10 of each; Table 4, Supplementary Table S8), and the top 10 GO terms and top 10 KEGG pathways of the DEGs are displayed in Figure 5C, Figure 5D, and Supplementary Table S8. Both upregulated and downregulated genes were involved in the regulation of transcription, DNA templates, and signal transduction as well as the positive regulation of transcription by RNA polymerase in the BP category. The upregulated genes also participated in immune and inflammatory responses. The KEGG results indicated that upregulated genes were enriched predominantly in pathways related to influenza A cytokine-cytokine receptor interaction, cell adhesion molecules (CAMs), *Staphylococcus aureus* infection, NOD-like receptors, chemokines and TNF signaling pathways. The downregulated genes were involved in pathways related to Hippo signaling, Wnt signaling, terpenoid backbone biosynthesis, proteoglycans in cancer, melanogenesis, bladder cancer, transcriptional misregulation in cancer, basal cell carcinoma, glycolysis/gluconeogenesis, and TGF-beta signaling.

**TABLE 4:** Top 10 upregulated and top 10 downregulated DEGs (CPB2 vs control).

Gene name	Locus	Regulation	Fold change	*p*-value
OLFM4	chr11:26,350,032–26,375,740	up	164.4964353	1.90719E-36
CCL17	chr6:19,345,764–19,348,965	up	60.09315203	4.09127E-21
HHATL	chr13:26,205,616–26,219,937	up	59.85677737	1.96714E-28
GC	chr8:68,326,287–68,364,937	up	57.73376422	3.20932E-22
PRICKLE2	chr13:45,620,368–45,789,113	up	43.95440161	1.42386E-18
C3	chr2:72,431,163–72,471,564	up	27.30318327	3.2955E-152
SLFN11	chr12:39,986,366–39,992,685	up	21.62979927	1.75657E-14
ADORA2A	chr14:49,468,862–49,487,628	up	14.17608216	1.33747E-29
PCP4L1	chr4:89,186,937–89,212,840	up	13.8161881	2.28515E-13
CCL22	chr6:19,296,637–19,302,737	up	11.84724721	1.74685E-16
ZSCAN26	chrX:45,772,497–45,798,482	down	0.137483812	3.34197E-27
ZFAND1	chr15:32,408,854–32,475,979	down	0.151873654	2.66575E-07
ITGA1	chr6:47,643,560–47,657,784	down	0.161195423	9.01136E-06
OCA2	chr7:22,114,800–22,133,148	down	0.18087438	1.64436E-12
NAB2	chr4:54,908,876–54,931,825	down	0.195968666	1.13013E-05
ZMAT2	chr16:32,185,484–32,295,491	down	0.19937155	0.012466794
ANKRD61	chr15:56,657,598–56,869,920	down	0.199594035	0.003131712
SPRY4	chr5:22,400,548–22,410,479	down	0.203847311	1.0527E-10
EGR1	chr2:142,411,086–142,417,154	down	0.209337433	9.52077E-24
MFAP3	chr3:5,097,791–5,117,889	down	0.210674837	3.27485E-06

### Combined m^6^A-Seq and RNA-Seq Analysis

**FIGURE 6:**
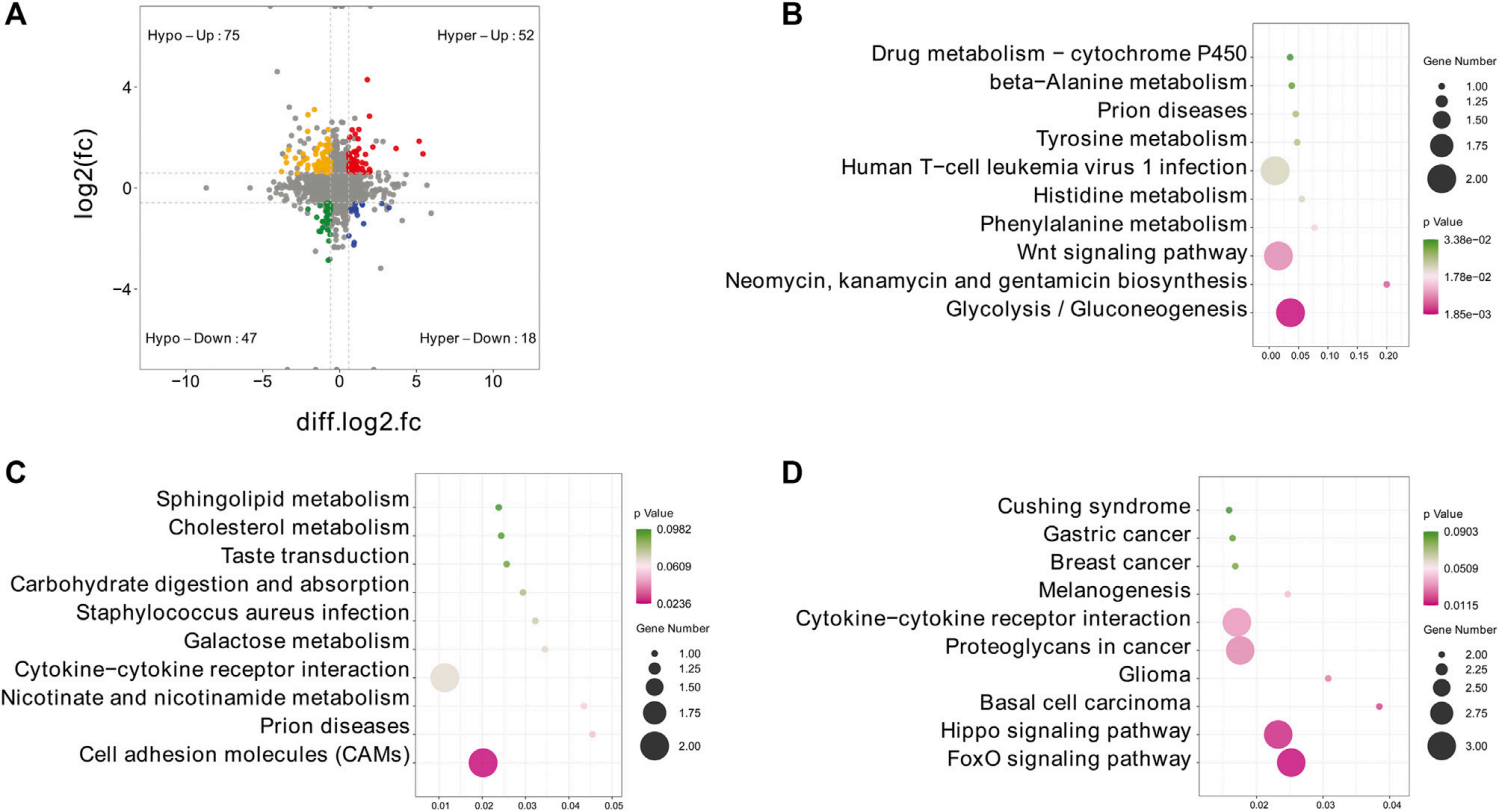
Combined m6A-seq and RNA-seq analysis. **(A)** Four-quadrant diagram depicting the distributions of DMGs and DEGs. **(B–D)** Top 10 significantly enriched KEGG pathways among the identified **(B)** hyper-down genes, **(C)** hyper-up genes and **(D)** hypo-up genes.

To further explore the functional significance of m^6^A modification in IPEC-J2 cells in response to CPB2, we investigated whether m^6^A methylation was the basis of the observed expression differences. For this purpose, m^6^A-seq data and RNA-seq data were used to detect DMGs and DEGs. Thereafter, combined m^6^A-seq and RNA-seq analysis divided a total of 192 genes into four main groups (Figure 6A and Supplementary Table S9): a group of 52 hypermethylated and upregulated genes (hyper-up genes), a group of 47 hypomethylated and downregulated genes (hypo-down genes), a group of 18 hypermethylated and downregulated genes (hyper-down genes) and a group of 75 hypomethylated and upregulated genes (hypo-up genes). The 18 hyper-down genes, 52 hyper-up genes and 75 hypo-up genes were further investigated by KEGG analysis, which revealed that the top 10 enriched KEGG pathways among the hyper-down genes were mainly related to glycolysis/gluconeogenesis; neomycin, kanamycin, gentamicin biosynthesis and Wnt signaling pathways (Figure 6B and Supplementary Table S9). In contrast, the hyper-up genes were mainly enriched in the CAMs signaling pathway (Figure 6C and Supplementary Table S9). In addition, the 75 hypo-up genes were mainly enriched in signaling pathways such as the FoxO, Hippo, and basal cell carcinoma pathways (Figure 6D and Supplementary Table S9).

### Validation of DEGs and DMGs by qPCR and MeRIP-qPCR

**FIGURE 7:**
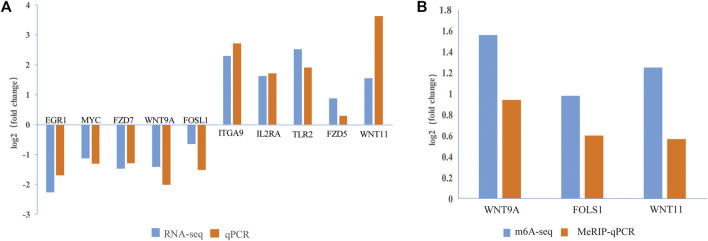
Verification of DEGs and DMGs. **(A)** The expression levels of 10 genes were verified by qPCR and RNA-seq. **(B)** The m^6^A methylation modifications of three genes were verified by MeRIP-qPCR and m^6^A-seq. The fold changes are expressed as the ratios of gene expression in CPB2-treated vs control samples. The blue and orange bars indicate the RNA-seq and m^6^A-seq or qRT-PCR and MeRIP-qPCR results, respectively.

To verify the m^6^A-seq and RNA-seq data, we randomly selected 10 DEGs (*EGR1*, *MYC*, *FZD7*, *WNT9A*, *FOSL1*, *ITGA9*, *IL2RA*, *TLR2*, *FZD5*, and *WNT11*) and checked the reliability of the RNA-seq data by RT-qPCR. According to the combined m^6^A-seq and RNA-seq analysis results, three DMGs (*WNT9A*, *FOSL1*, and *WNT11*) were selected for the MeRIP-qPCR assay. The RT-qPCR and MeRIP-qPCR results (Figures 7A,B) were consistent with the RNA-seq and m^6^A-seq results, thus confirming the reliability of the results of our m^6^A-seq experiment.

## Discussion

Similar to DNA and protein modifications, m^6^A modification is a dynamically reversible posttranscriptional change that mainly regulates mRNA expression, splicing, structure, stability, lifespan, and degradation as well as RNA modification during translation (Fu et al., 2014; Adhikari et al., 2016). This modification plays roles in bacterial and viral infections, intestinal diseases, and host immune responses (Sebastian-delaCruz et al., 2020; Wu et al., 2020). Feng et al. (2018) demonstrated that the expression levels of m^6^A and *METTL3* were upregulated in HDPCs stimulated by LPS. Similarly, in another study, both the m^6^A levels and the mRNA expression of *WTAP* increased when THP-1 cells were treated with heat-killed *Salmonella typhimurium* (HKST) (Wu et al., 2020). However, Zong et al. found that when IPEC-J2 cells were infected with *E. coli* K88, the methylation level of m^6^A was significantly reduced, while the expression of m^6^A reader proteins (*YTHDF1* and *YTHDF2*) were markedly increased, and the expression of the methyltransferase *METTL3* and the demethylase *FTO* were not changed; moreover, *E. coli* K88 infection was shown to cause intestinal inflammation and to impair lipid transport in the *YTHDF1*-dependent m^6^A pathway in IPEC-J2 cells (Zong et al., 2018). In this experiment, we detected the mRNA expression of methylation-related enzymes and the overall level of m^6^A methylation, and the results demonstrated that the mRNA expression levels of the methyltransferases *METTL3*, *METTL14*, and *WTAP* and the demethylases *FTO* and *ALKBH5* were altered, while the overall m^6^A level was increased significantly. Therefore, we speculate that the m^6^A methylation modification may be involved in the CPB2-induced inflammatory response of IPEC-J2 cells.

In this study, we found 18,695 peaks that were the same in the CPB2 and control groups, which accounted for 90% of all the peaks. This indicated that most of the peaks corresponded to functions that maintained the physiological homeostasis of the organism. In mouse tissues and human cells, m^6^A has been shown to be located mainly near stop codons and 3′UTRs (Dominissini et al., 2012; Meyer et al., 2012). We analyzed the distributions of m^6^A peaks in different gene functional elements and found that m^6^A peaks were indeed mainly distributed in 3′UTRs and stop codon regions. These findings are consistent with those of Wang et al. (2020b) and prove that m^6^A motif enrichment is more common in 3′UTRs than in other regions. The 3′UTR regulates the stability, localization, expression, and translation of mRNA. Multiple RNA-binding proteins bind in this region to perform regulatory functions and regulate protein–protein interactions (Mayr, 2018). The m6A methylation recognition proteins, YTHDF1 and YTHDF2, mainly recognize the m^6^A motifs of 3′UTRs and alter the translation efficiency and degradation rate of m^6^A-modified RNA (Wang et al., 2015; Chen et al., 2018). A conserved m^6^A motif sequence, gg (R: G, A, U; R: G, A; H: U, A, C), was recently reported (Han et al., 2019). Our results revealed conserved m^6^A motifs of GGACU and UGGACU in the control and CPB2 groups, respectively, similar to the findings of Han et al. (2019). These results suggest that these motifs may be recognized by certain proteins and play important roles in IPEC-J2 cells in response to CPB2.

Relative to the control group, the CPB2 group exhibited 1,448 differential peaks (437 upregulated and 1,011 downregulated). The DMGs with upregulated peaks were mainly enriched in cancer signaling pathways, the Cushing syndrome signaling pathway and the Wnt signaling pathway. Interestingly, some of these genes appeared simultaneously in many signaling pathways. For example, *WNT11*, *WNT9A*, and *FZD9* were all enriched in cancer signaling pathways, the Cushing syndrome signaling pathway, and the Wnt signaling pathway. These three genes encode the main ligands and receptors that regulate the activation and deactivation of the Wnt signaling pathway. The Wnt signaling pathway impacts intestinal balance, self-renewal, and malignant transformation (Liu et al., 2011). The *WNT11* gene is a novel important contributor to intestinal homeostasis and host defense and participates in the protection of host intestinal cells by blocking the invasion of pathogenic bacteria, inhibiting inflammation, and inhibiting apoptosis (Liu et al., 2011). In addition, some studies have indicated that *WNT11* regulates the development of the heart and kidneys via an atypical Wnt signaling pathway and inhibits the inflammation of intestinal epithelial cells (Pandur et al., 2002; Majumdar et al., 2003; Liu et al., 2011). *WNT9A* is considered to act as a tumor suppressor gene in relation to the development of colorectal cancer (Ali et al., 2016). Intriguingly, according to the GO and KEGG analyses, we found that the DMGs and DEGs were almost always significantly enriched in the Wnt signaling pathway., Therefore, we propose that m^6^A modification is involved in the inflammatory response induced by CPB2 in IPEC-J2 cells and that it may modulate this inflammatory response by influencing the gene expression of the Wnt signaling pathway.

## Conclusion

In this study, we analyzed how m^6^A methylation is modified in CPB2-induced IPEC-J2 cells. The results suggest that m^6^A methylation may play a role in the Wnt signaling pathway in CPB2-induced IPEC-J2 cells and exert anti-inflammatory or proinflammatory effects on intestinal diseases. These findings provide a basis for further research into the functions of m^6^A methylation modifications in CPB2 toxin-induced piglet diarrhea.

## Data Availability

Our data are deposited in the G E O repository, the accession number: GSE167267. We have published our data on October 5, 2021. The link for data disclosure is as follows: https://www.ncbi.nlm.nih.gov/geo/query/acc.cgi?acc=GSE167267.
