# Supplementary figures and images for: Comprehensive Analysis of Transcriptome-wide m6A Methylome Upon Clostridium perfringens Beta2 Toxin Exposure in Porcine Intestinal Epithelial Cells by m6A Sequencing
Source: Front Genet. 2021 Oct 19;12:689748. doi: 10.3389/fgene.2021.689748 (PMC8560698; doi:10.3389/fgene.2021.689748)

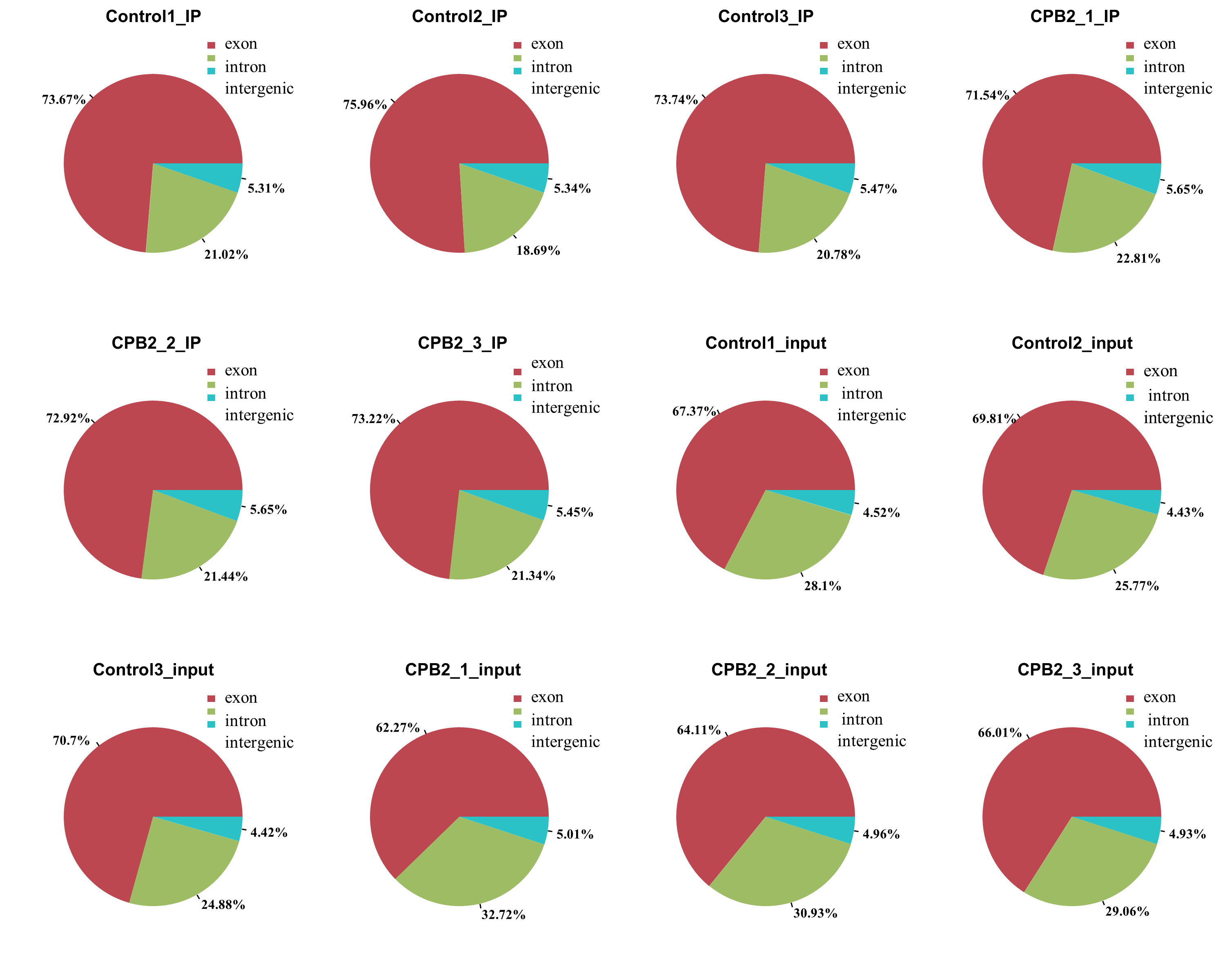

Supplement: Supplementary file 4 [file Image1.TIF]
